# Multidrug resistance pattern and molecular epidemiology of pathogens among children with diarrhea in Bangladesh, 2019–2021

**DOI:** 10.1038/s41598-023-41174-6

**Published:** 2023-08-26

**Authors:** Nadim Sharif, Shamsun Nahar Ahmed, Shamim Khandaker, Nuzhat Haque Monifa, Ali Abusharha, Debora Libertad Ramírez Vargas, Isabel De la Torre Díez, Angel Gabriel Kuc Castilla, Ali Azam Talukder, Anowar Khasru Parvez, Shuvra Kanti Dey

**Affiliations:** 1 Department of Microbiology, Jahangirnagar University, Savar, Dhaka, 1342 Bangladesh; 2 Optometry Department. Applied Medical Sciences Collage, King Saud University, P. O. Box 145111, Riyadh, Saudi Arabia; 3 Universidad Internacional Iberoamericana, 24560 Campeche, Mexico; 4 University of Valladolid, Valladolid, Spain; 5 Universidad Europea del Atlántico, Isabel Torres 21, 39011 Santander, Spain

**Keywords:** Antimicrobial resistance, Bacteria, Infectious-disease diagnostics, Diseases

## Abstract

Antimicrobial and multidrug resistance (MDR) pathogens are becoming one of the major health threats among children. Integrated studies on the molecular epidemiology and prevalence of AMR and MDR diarrheal pathogens are lacking. A total of 404 fecal specimens were collected from children with diarrhea in Bangladesh from January 2019 to December 2021. We used conventional bacteriologic and molecular sequence analysis methods. Phenotypic and genotypic resistance were determined by disk diffusion and molecular sequencing methods. Fisher’s exact tests with 95% confidence intervals (CIs) was performed. Prevalence of bacterial infection was 63% (251 of 404) among children with diarrhea. *E. coli* (29%) was the most prevalent. *E. coli*, *Shigella* spp., *V. cholerae*, and *Salmonella* spp., showed the highest frequency of resistance against ceftriaxone (75–85%), and erythromycin (70–75%%). About 10–20% isolates of *E. coli*, *V. cholerae* and *Shigella* spp. showed MDR against cephem, macrolides, and quinolones. Significant association (*p* value < 0.05) was found between the phenotypic and genotypic resistance. The risk of diarrhea was the highest among the patients co-infected with *E. coli* and rotavirus [OR 3.6 (95% CI 1.1–5.4) (*p* = 0.001)] followed by *Shigella* spp. and rotavirus [OR 3.5 (95% CI 0.5–5.3) (*p* = 0.001)]. This study will provide an integrated insight of molecular epidemiology and antimicrobial resistance profiling of bacterial pathogens among children with diarrhea in Bangladesh.

## Introduction

Diarrheal diseases are the second major cause of mortality among children under five years of age^1^. Nearly 500,000 children aged under five years including about 50,000 neonates die annually due to diarrheal diseases^1–3^. Every year an estimated 2 billion cases of diarrhea occur among children globally^1–4^.

Diarrheagenic bacteria significantly contributes to the etiology of gastroenteritis among children^2–4^. The proportionate incidence of different bacterial pathogens may vary in different geographical regions. However, prevalence of *V.cholerae, Shigella* spp., enterotoxigenic *E. coli*, and *Salmonella* spp. are consistently documented globally^1–6^.

Every year about 35,000 deaths are reported among children with diarrheal diseases in Bangladesh^3–7^. However, due to lack of strong surveillance and health system majority of the cases remains unreported in Bangladesh. In less than 5% of cases the pathogen is accurately investigated and identified^3^. Improper diagnosis leads to inappropriate use of antibiotics against diarrheal diseases in Bangladesh^3,8,9^.

The selection pressure of antimicrobial resistance strains has been increased and effectiveness of antimicrobials have decreased due to abuse of antibiotics^10,11^. Further, horizontal transfer of resistance genes within different bacterial pathogens have contributed to origin and dissemination of multidrug resistance (MDR) phenomenon and strains with extended-spectrum β-lactamases (ESBL) in environment, animals and humans^10–13^. Antibiotic-resistant bacteria have been found all over the world in common bacterial illnesses including diarrhea^13^. Unfortunately, majority of the bacterial pathogens associated with diarrhea haven been found developing antimicrobial resistance worldwide^13–19^. Recent studies in Bangladesh have reported increased incidence of multi-drug resistance *E coli*, *Salmonella* spp. and *Shigella* spp. in different human and environmental samples. The prevalence of diarrheagenic bacteria is high among children in Bangladesh^3,13–15^. Multidrug resistance bacterial pathogens in children can contribute to prolonged hospitalization and treatment failure^13–15^. Being a major health threat, antibiotic resistance leads to higher medical costs, prolonged hospital stays, increased morbidity and increased mortality^13,14^. Presence of antimicrobial and multidrug resistance bacterial infection can add to the existing health burden of diarrheal diseases in Bangladesh. However, studies on the multidrug resistance profiling of these major diarrheal pathogens in Bangladesh are not enough, which is a major public health concern.

Integrated study on the epidemiology, antibiotic resistance profiling and determining the association between phenotypic and genotypic resistance of bacterial pathogens isolated from children with diarrhea are lacking. The main aim of this study was to investigate the molecular epidemiology of bacterial pathogens associated with diarrheal diseases among children in Bangladesh. Further, this study was conducted to evaluate the prevalence of antimicrobial resistance and multidrug resistance properties of bacterial isolates among diarrheal children, their molecular markers and association of genotypic and phenotypic resistance of pathogens.

## Results

### Demographic characteristics of the pediatric patients

The ratio of male to female children was 2:1 (62% and 38%, respectively). About 43% (173 of 404) of the children with diarrhea aged below 12 months. Majority of the patients (80%) were from village areas. Nearly 90% (362 of 404) of the pediatric patients with diarrhea required hospitalization and 24% (98 of 404) showed prolonged infection for ≥ 6 days. Antibiotics were used in treatment of 95.5% of the children with diarrhea (Table 1).

**Table 1:** Risk of infection by different diarrheal pathogens among children according to their demographic conditions.

Variables	Case patients no./total no. (%)	Risk of infection by bacterial pathogens (95% CI) (*p* value)	Risk of infection by virus pathogens (95% CI) (*p* value)	Risk of mixed infection (95% CI) (*p* value)
Sex				
Male	248/404 (62)	1.85 (0.97–2.64) (.0001)	1.54 (0.84–1.9) (.01)	1.86 (0.72–2.9) (.003)
Female	156/404 (38)	1.1 (0.32–1.5) (.005)	0.56 (0.2–0.64) (.002)	1.02 (0.42–1.95) (.005)
Age in months				
1–3	93/404 (23)	2.3 (0.9–2.8) (.01)	5.85 (2.3–7.42) (.005)	1.6 (0.53–2.77) (.03)
4–11	80/404 (20)	1.85 (0.73–2.95) (.005)	3.59 (1.85–4.98) (.003)	1.85 (0.62–3.4) (.004)
12–23	75/404 (18.5)	1.5 (0.6–2.7) (.002)	2.1 (0.3–3.9) (.001)	1.02 (0.4–1.8) (.03)
24–35	64/404 (16)	2.1 (0.9–3.4) (.001)	3.53 (1.32–5.65) (.003)	1.54 (0.6–2.4) (.01)
36–47	43/404 (11)	1.15 (0.4–2.64) (.001)	1.3 (0.4–2.5) (.003)	1.6 (0.7–2.9) (.004)
48–60	28/404 (7)	1.52 (0.45–3.85) (.001)	1.93 (0.62–2.85) (.003)	1.45 (0.4–2.8) (.001)
> 60	21/404 (5.2)	0.4 (0.2–1.8) (.006)	1.6 (0.6–2.5) (.001)	1.89 (0.22–2.95) (.005)
Hospitalization				
≤ 2 days	78/404 (19)	1.3 (0.66–1.94) (.03)	2.68 (1.32–3.89) (.007)	0.8 (0.2–1.9) (.07)
3–5 days	186/404 (46)	1.81 (0.87–2.95) (.005)	3.23 (1.48–4.93) (.001)	2.63 (1.57–3.85) (.003)
≥ 6 days	98/404 (24)	3.87 (1.65–5.74) (.001)	1.05 (0.22–1.98) (.005)	4.5 (2.8–7.55) (.005)
Not hospitalized	42/404 (10)	1.42 (0.53–1.95) (.006)	0.8 (0.2–1.7) (.03)	0.6 (0.1–1.3) (.05)
Antibiotics used				
Yes	386/404 (95.5)	3.42 (1.52–5.82) (.005)	1.01 (0.86–1.74) (.84)	1.86 (0.53–2.21) (.003)
No	18/404 (4.5)	0.21 (0.1–0.95) (.53)	0.73 (0.32–1.87) (.64)	0.91 (0.38–1.24) (.004)

### Prevalence of bacterial pathogens among children

Nearly 63% (251 of 404) fecal specimens were tested positive for bacterial pathogens. Among bacterial pathogens detected, *E. coli* was the most prevalent (29.2%, 117 of 404) followed by *Shigella* spp. (17%, 68 of 404), *V. cholerae* (13.2%, 53 of 404), and *Salmonella* spp. (5.5%, 22 of 404), respectively (Supplementary Fig. i). We found a seasonal peak for infection of bacterial pathogens during May and August for 2019, 2020 and 2021 (Supplementary Fig. ii).

### Analysis of mixed infection in children

The most prevalent mixed infection of children was caused by rotavirus and *E. coli* (15%, 60 of 404), followed by *V. cholerae* and *Shigella* spp. (7.7%, 31 of 404), (Table 2). Rotavirus was the major co-infecting virus (Supplementary Fig. iii). Co-infection with two pathogens was most prevalent (31%, 127 of 404) followed by three pathogens (12%, 48 of 404) and four pathogens (8%, 32 of 404), respectively.

**Table 2:** Risk analysis for disease outcome among children infected with mono-pathogens and co-pathogens of diarrhea.

Pathogens	Co-infecting pathogens	Case patient no/total no. (%)	Diarrhea	Vomiting	Fever	Dehydration	Abdominal pain
*E. coli*		117/404 (29)	2.3 (0.9–3.8) (.001)	2.1 (0.5–3.5) (.003)	1.7 (0.6–3.8) (.005)	1.4 (0.5–2.9) (.08)	0.8 (0.1–2.) (.05)
	Rotavirus (RV)	60/404 (15)	3.6 (1.1–5.4) (.001)	1.3 (0.7–3.8) (.05)	1.2 (0.3–2.9) (.07)	2.6 (1.1–3.9) (.005)	1.6 (0.6–3.5) (.08)
	Norovirus (NoV)	11/404 (3)	2.2 (0.8–4.6) (.001)	3.8 (1.6–5.4) (.0003)	0.9 (0.1–1.7) (.005)	2.8 (0.8–3.8) (.05)	1.4 (0.2–3.1) (.004)
	Adenovirus (AdV)	6/404 (1.5)	1.2 (0.6–2.8) (.005)	.9 (0.1–2.8) (.008)	0.8 (0.1–2.6) (.003)	1.9 (0.8–3.5) (.005)	1.1 (0.1–2.5) (.002)
	Both RV and NoV	8/404 (2)	3.5 (1.3–6.8) (.002)	1.8 (0.5–3.5) (.05)	1.3 (0.2–2.1) (.008)	1.6 (0.5–2.8) (.05)	2.3 (1.2–4.5) (.005)
	*V. cholerae*	18/404 (4.5)	1.5 (0.8–3.8) (.002)	1.4 (0.6–3.3) (.005)	1.1 (0.5–2.9) (.006)	2.1 (0.6–3.8) (.003)	0.6 (0.08–1.1) (.08)
	*Shigella* spp.	12/404 (3)	1.3 (0.6–3.8) (.01)	1.2 (0.4–3.1) (.003)	2.4 (0.8–4.8) (.05)	1.5 (0.5–2.6) (.0001)	1.6 (0.7–3.8) (.005)
	*Salmonella* spp.	5/404 (1.2)	1.8 (0.5–3.6) (.001)	1.5 (0.3–3.8) (.005)	1.7 (0.5–3.6) (.03)	2.4 (1.4–4.2) (.005)	1.2 (0.1–3.2) (.05)
*V. cholerae*		53/404 (13)	2.5 (1.1–4.6) (.005)	1.2 (0.6–2.8) (.008)	1.8 (0.9–3.2) (.01)	1.4 (0.4–2.9) (.08)	0.8 (0.2–2.9) (.05)
	Rotavirus	21/404 (5)	2.8 (1.4–4.5) (.003)	1.5 (0.2–2.8) (.04)	0.4 (0.05–1.1) (.001)	1.5 (0.9–2.3) (.03)	1.2 (0.5–3.1) (.006)
	Norovirus	2/404 (0.5)	1.5 (0.5–3.5) (.001)	2.6 (1.4–4.5) (.03)	1.2 (0.3–3.5) (.007)	1.9 (0.8–3.7) (.001)	1.3 (0.5–3.8) (.005)
	Both RV and NoV	1/404 (0.25)	2.8 (1.2–4.5) (.006)	1.5 (0.4–3.8) (.002)	2.4 (1.2–3.9) (.003)	0.2 (0.08–1.6) (.09)	1.6 (0.2–3.9) (.002)
	*Shigella* spp.	31/404 (7.7)	1.2 (0.1–2.8) (.007)	1.6 (0.2–3.7) (.01)	1.3 (0.2–3.8) (.04)	0.8 (0.2–2.3) (.01)	1.3 (0.5–2.9) (.08)
	*Salmonella* spp.	15/404 (3.8)	1.1 (0.2–2.5) (.05)	2.5 (0.5–4.8) (.002)	2.1 (1.3–4.6) (.001)	1.3 (0.6–2.3) (.005)	1.8 (0.5–3.5) (.002)
*Shigella* spp.		68/404 (17)	1.8 (0.6–2.9) (.001)	2.4 (0.9–4.7) (.01)	1.3 (0.4–3.9) (.005)	1.5 (0.3–3.8) (.00001)	1.3 (0.2–3.2) (.05)
	Rotavirus	19/404 (4.7)	3.5 (0.5–5.3) (.001)	1.9 (0.9–3.6) (.005)	1.9 (0.6–3.9) (.06)	1.3 (0.5–3.2) (.00001)	0.8 (0.1–3.8) (.005)
	Norovirus	3/404 (0.75)	1.6 (0.8–3.8) (.005)	1.2 (0.2–2.9) (.01)	1.6 (0.5–4.1) (.002)	1.3 (0.2–2.8) (.05)	0.7 (0.97–2.64) (.0001)
	Both RV and NoV	2/404 (0.5)	1.5 (0.6–3.5) (.003)	1.5 (0.7–3.2) (.005)	1.3 (0.5–3.8) (.001)	1.8 (0.9–3.8) (.0001)	1.6 (0.3–5.5) (.06)
	*Salmonella* spp.	11/404 (2.7)	1.2 (0.2–2.9) (.03)	1.1 (0.6–3.8) (.001)	0.2 (0.06–1.2) (.08)	2.4 (1.2–5.9) (.005)	1.3 (0.2–3.8) (.002)
*Salmonella* spp.		22/404 (5.5)	2.1 (0.5–4.5) (.01)	1.4 (0.3–4.2) (.05)	1.6 (0.4–3.1) (.02)	1.1 (0.6–3.6) (.01)	0.2 (0.1–1.5) (.003)
	Rotavirus	7/404 (1.7)	1.6 (0.8–3.8) (.008)	1.1 (0.1–2.7) (.003)	1.2 (0.4–3.5) (.02)	1.6 (0.7–3.8) (.003)	1.8 (0.9–4.2) (.08)
	Norovirus	2/404 (0.5)	1.6 (0.9–2.9) (.8)	1.7 (0.6–3.8) (.002)	0.7 (0.1–2.2) (.003)	1.9 (0.6–3.6) (.001)	0.3 (0.1–2.9) (.006)
No pathogen detected		153/404 (37)	0.8 (0.1–1.1) (.03)	.3 (0.1–1.1) (.005)	1.8 (0.7–2.8) (.01)	0.5 (0.1–1.3) (.02)	.2 (0.1–0.9) (.002)

### Risk of infections by bacteria and viruses among children

Male patient had a higher risk of bacterial [OR 1.85 (95% CI 0.97–2.64) (*p* = 0.0001)], viral [OR 1.54 (95% CI 0.84–1.9) (*p* = 0.01)] and mixed infection [OR 1.86 (95% CI 0.72–2.9) (*p* = 0.003)] than female patients. Children in all age groups were more susceptible to infection of virus than bacteria or mixed infection. The odds ratio of infection by virus was the highest [OR 5.85 (95% CI 2.3–7.42) (*p* = 0.005)] in children aged 1–3 months followed by 4-11 months [OR 3.59 (95% CI 1.85–4.98) (*p* = 0.003)]. The risk of bacterial infection was also higher in children aged 1–3 months [OR 2.3 (95% CI (0.9–2.8) (*p* = 0.01)] followed by 24–35 months [OR 2.1 (95% CI 0.9–3.4) (*p* = 0.001)]. However, the risk of mixed infection was the highest [OR 1.89 (95% CI 0.22–2.95) (*p* = 0.005)] in children aged above 60 months (Table 1). The risks of infection by different pathogens among the hospitalized children were also analyzed. Children hospitalized for ≥ 6 days had higher risk of bacterial infection [OR 3.87 (95% CI 1.65–5.74) (*p* = 0.001)] and co-infection [OR 4.5 (95% CI 2.8–7.55) (*p* = 0.005)] (Table 1).

### Clinical features associated with diarrheal diseases

Diarrhea (87%, 351 of 404) was most common followed by abdominal pain (82%, 331 of 404), vomiting (73%, 295 of 404), dehydration (68%, 274 of 404) and fever (59%, 238 of 404) among the pediatric patients. Among the children infected with *E. coli*, diarrhea (74%) was the most common symptoms followed by fever (65%), (Supplementary Fig. iv). Similarly, among children with *V. choleare* infection, abdominal pain (76%) was the most prevalent symptom. Apparently, the most common duration of diarrhea was 5 days among children infected with *E. coli* (Supplementary Fig. v).

### Association of coinfection with health outcomes among patients with diarrhea

The risk of diarrhea was the highest among the patients co-infected with *E. coli* and rotavirus [OR 3.6 (95% CI 1.1–5.4) (*p* = 0.001)] followed by *Shigella* spp. and rotavirus [OR 3.5 (95% CI 0.5–5.3) (*p* = 0.001)], and triple infection of *E. coli*, rotavirus and norovirus [OR 3.5 (95% CI 1.3–6.8) (*p* = 0.002)], respectively (Table 2). The risk of vomiting was the highest in children with co-infection of *E. coli* and norovirus [OR 3.8 (95% CI 1.6–5.4) (*p* = 0.0003)]. In mono-infection, children with infection of *V. cholerae* had higher risk of diarrhea [OR 2.5 (95% CI 1.1–4.6) (*p* = 0.005)] and fever [OR 1.8 (95% CI 0.9–3.2) (*p* = 0.01)] (Table 2).

### Frequency of antimicrobial resistance of bacterial pathogens

**Figure 1:**
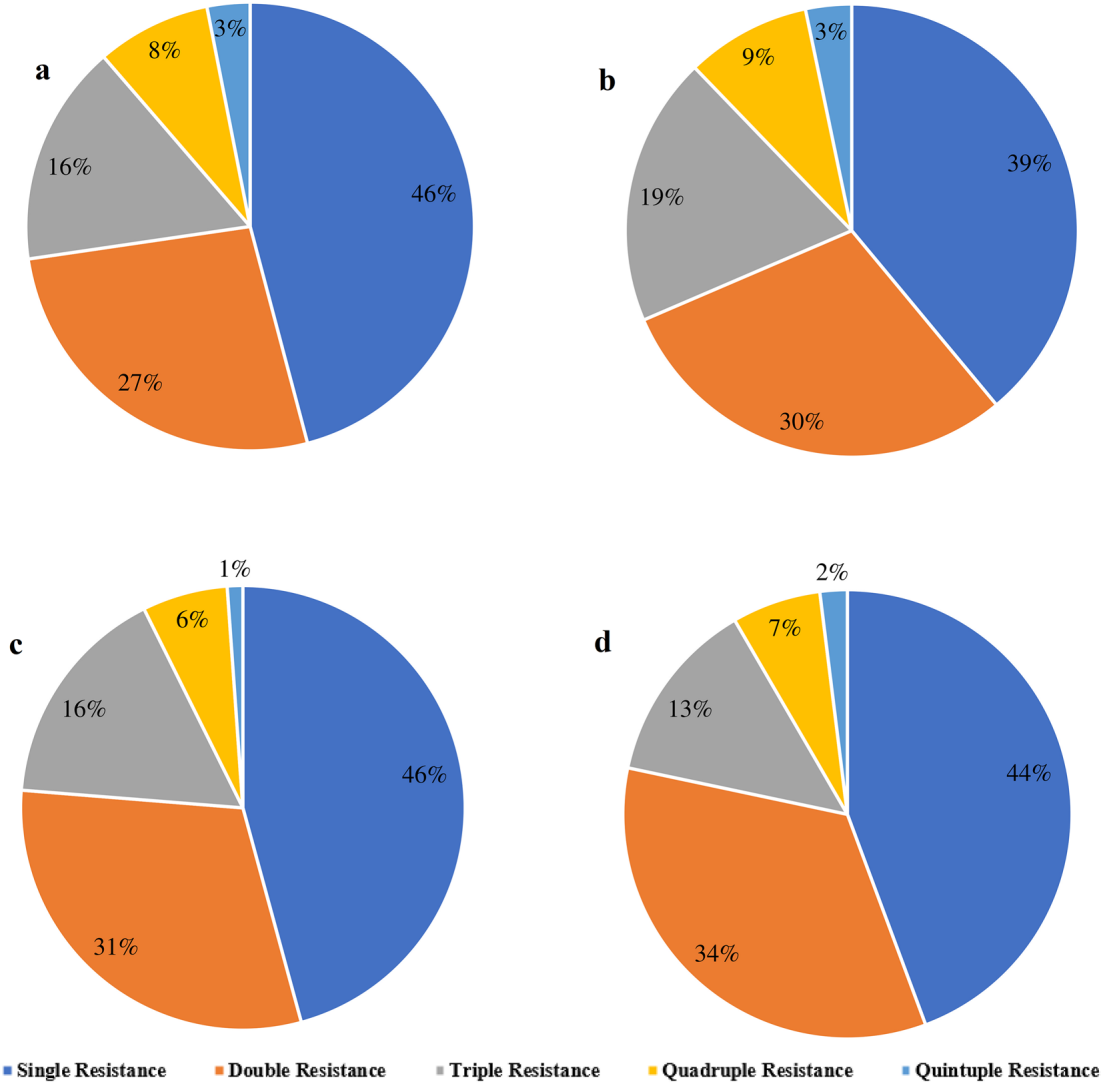
Proportionate percentage of single, double, triple, quadruple and quintuple resistance isolates of (**a**) *Escherichia coli*, (**b**) *Vibrio cholerae*, (**c**) *Salmonella* spp. and (**d**) *Shigella* spp.

Among the isolates of *E. coli,* the highest frequency of resistance was detected against ceftriaxone (79%) and erythromycin (79%) followed by norfloxacin (62%), ciprofloxacin (58%), and tetracycline (52%), respectively. On the contrary, most of the isolates of *E. coli* (70%) were sensitive against imipenem followed by colistin (53%) (Table 3). Isolates of *V. cholerae* showed highest resistance against erythromycin (81%). Similar to *E coli*, *V. cholerae* showed the highest sensitivity against imipenem (64%) and colistin (59%). Most of the isolates of *Salmonella* spp. showed resistance against erythromycin (86%) and ceftriaxone (77%). Besides colistin, isolates of *Salmonella* spp. showed high frequency of sensitivity against meropenem (59%) and imipenem (Table 3). Majority of the isolates of *Shigella* spp. showed resistance against ceftriaxone (88%), erythromycin (76%), and norfloxacin (72%). Isolates of *E. coli* (16%, 19 of 117), *V. cholerae* (19%, 10 of 53) and *Shigella* spp. (13%, 9 of 68) showed multidrug resistance against antibiotics of cephem (ceftriaxone), macrolides (erythromycin), quinolones (norfloxacin) and phenicol (chloramphenicol) (Fig. 1). Single resistance was most prevalent among the isolates of *E. coli* (46%)*, V. cholerae* (39%) *Salmonella* spp. (46%) and *Shigella* spp. (44%) (Fig. 1). About 22–31% of the isolates of bacterial pathogens showed resistance against three or more groups of antibiotics (Fig. 1).

**Table 3:** Antimicrobial resistance profiling of isolates of bacterial pathogens detected from children with diarrhea.

Antibiotics groups	Antibiotics	Classification	*E. coli* n = 117 number of isolates (%)	*V. cholera* n = 53 number of isolates (%)	*Salmonella* spp. n = 22 number of isolates (%)	*Shigella* spp. n = 68 number of isolates (%)
Cephem	CRO	Resistant	92 (79)	41 (77)	17 (77)	60 (88)
		Intermediate	3 (2)	4 (8)	2 (9)	4 (6)
		Sensitive	22 (19)	8 (15)	3 (14)	4 (6)
Carbapenems	IPM	Resistant	19 (16)	8 (15)	4 (18)	11 (17)
		Intermediate	16 (14)	11 (21)	5 (23)	18 (25)
		Sensitive	82 (70)	34 (64)	13 (59)	39 (57)
	MEM	Resistant	12 (10)	9 (17)	7 (32)	17 (25)
		Intermediate	47 (40)	17 (32)	4 (18)	12 (18)
		Sensitive	58 (50)	27 (51)	11 (50)	39 (57)
Macrolides	E	Resistant	92 (79)	43 (81)	19 (86)	52 (76)
		Intermediate	17 (14)	7 (13)	2 (9)	12 (18)
		Sensitive	8 (7)	3 (6)	1 (5)	4 (6)
Tetracycline	TE	Resistant	61 (52)	20 (38)	9 (41)	17 (25)
		Intermediate	22 (19)	19 (36)	6 (27)	20 (29)
		Sensitive	34 (29)	14 (26)	7 (32)	31 (46)
	DO	Resistant	46 (39)	23 (43)	10 (45)	32 (47)
		Intermediate	16 (14)	12 (23)	5 (23)	12 (18)
		Sensitive	55 (47)	18 (34)	7 (32)	24 (35)
Quinolones	CIP	Resistant	68 (58)	27 (51)	12 (55)	38 (56)
		Intermediate	19 (16)	14 (26)	4 (18)	17 (25)
		Sensitive	30 (26)	12 (23)	6 (27)	13 (19)
	NOR	Resistant	72 (62)	33 (62)	15 (68)	49 (72)
		Intermediate	27 (23)	9 (17)	2 (9)	8 (12)
		Sensitive	18 (15)	11 (21)	5 (23)	11 (16)
Phenicol	CL	Resistant	60 (52)	30 (56)	13 (59)	42 (62)
		Intermediate	25 (21)	11 (21)	2 (9)	9 (13)
		Sensitive	32 (27)	12 (23)	7 (32)	17 (25)
Lipopeptide	C	Resistant	33 (28)	14 (26)	5 (23)	19 (28)
		Intermediate	22 (19)	8 (15)	7 (32)	15 (22)
		Sensitive	62 (53)	31 (59)	12 (55)	34 (50)
Folate pathway antagonist	COT	Resistant	53 (45)	23 (44)	10 (45)	36 (53)
		Intermediate	33 (29)	15 (28)	3 (14)	8 (12)
		Sensitive	31 (26)	15 (28)	9 (41)	24 (35)

*CRO* Ceftriaxone, *IPM* Imipenem, *MEM* Meropenem, *E* Erythromycin, *TE* Tetracycline, *DO* Doxycycline, *CIP* Ciprofloxacin, *NOR* Norfloxacin, *CL* Chloramphenicol, *C* Colistin, *COT* Cotrimoxazole.

### Association of phenotypic resistance with genotypic resistance elements

We detected resistance genes against quinolones (*qnr* genes), β-Lactams (*bla*_TEM_), folate pathway antagonist (Cotrimoxazole-*sxt* genes), tetracycline (*tet* genes) and colistin (*mcr* genes). Genetic resistance elements against ciprofloxacin, cotrimoxazole and colistin were found in lower frequency among the isolates of *E. coli* (6–11%), *V. cholerae* (7–13%), *Salmonella* spp. (9–14%) and *Shigella* spp. (9–15%) (Table 4 and Supplementary Fig. vi). Among the isolates of *E. coli, V. cholerae*, and *Salmonella* spp. the highest frequency of resistance genes was found against β-Lactams (36%, 42 of 117; 36%, 19 of 53; 45%, 10 of 22, respectively) followed by tetracyclines (18%, 21 of 117; 20%, 11 of 53; 27%, 6 of 22, respectively).

**Table 4:** Association of phenotypic and genotypic resistance of bacterial pathogens isolated from pediatric patients with diarrhea.

Pathogens (n)	CIP number of isolates (%)			CRO number of isolates (%)			C number of isolates (%)			COT number of isolates (%)			TE number of isolates (%)		
	Phen	Gen	*p-*value	Phen	Gen	*p-*value	Phen	Gen	*p-*value	Phen	Gen	*p-*value	Phen	Gen	*p-*value
*E. coli* (117)	68 (58)	12 (10)	0.037	92 (79)	42 (36)	0.001	33 (28)	7 (6)	0.02	53 (45)	11 (9)	0.002	61 (52)	21 (18)	0.009
*V. cholera* (53)	27 (51)	7 (13)	0.005	41 (77)	19 (36)	0.83	14 (26)	4 (7)	0.008	23 (43)	6 (11)	0.005	20 (38)	11 (20)	0.047
*Salmonella* spp. (22)	12 (55)	3 (14)	0.0004	17 (77)	10 (45)	0.03	5 (23)	3 (14)	0.36	10 (45)	5 (9)	0.86	9 (41)	6 (27)	0.02
*Shigella* spp. (68)	38 (56)	8 (12)	0.043	60 (88)	28 (41)	0.01	19 (28)	6 (9)	0.003	36 (53)	10 (15)	0.04	17 (25)	9 (13)	0.93

*Phen* Phenotypic, *Gen* Genotypic.

Significant association was found between the phenotypic resistance and genotypic elements among the isolates of *V. cholerae* (*p* = 0.005), and *Salmonella* spp. (*p* = 0.0004) against ciprofloxacin. Among the isolates of *E. coli* (*p* = 0.001), *Salmonella* spp. (*p* = 0.03) and *Shigella* spp. (*p* = 0.01) we found significant association between the phenotypic resistance and genotypic elements against ceftriaxone. Further, presence of the gene *mcr-1* was significantly associated with the resistance properties of isolates of *V. cholerae* (*p* = 0.008) and *Shigella* spp. (*p* = 0.003) against colistin. In addition, the presence of *sxt* gene among the isolates of *E. coli* (*p* = 0.002) and *V. cholerae* (*p* = 0.005) was significantly associated with their phenotypic resistance against cotrimoxazole (Table 4).

### Nucleotide sequence and phylogenetic analyses

**Figure 2:**
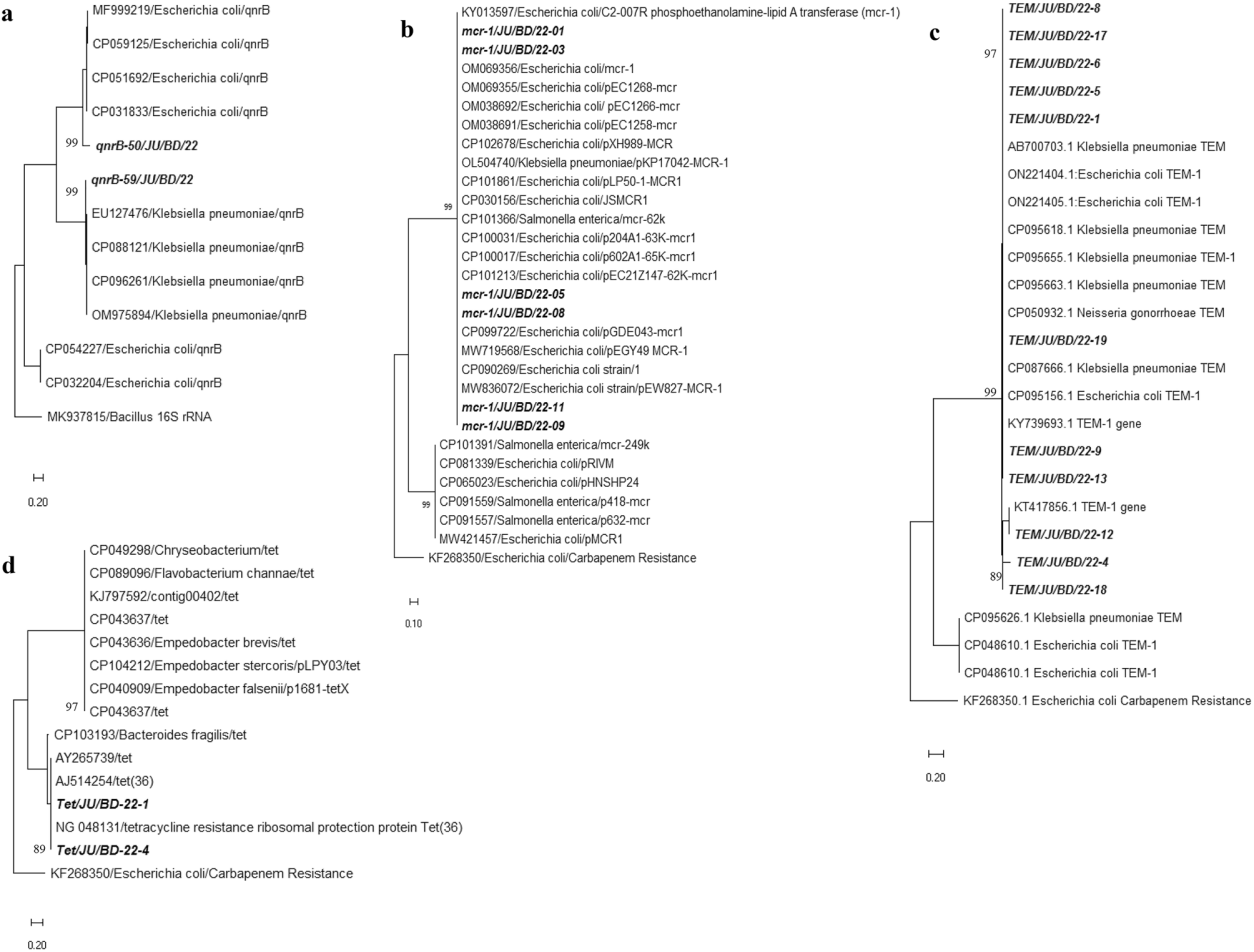
Phylogenetic relationship of antimicrobial resistance genes isolated from bacterial pathogens. (**a**) Partial sequences of *qnrB* gene from *E. coli* were used for constructing this tree, (**b**) Partial sequences of *mcr-1* gene from *E. coli* were used for constructing this tree, (**c**) Partial sequences of *bla*_*TEM*_ gene from *E. coli* were used for constructing this tree, (**d**) Partial sequences of *tetA* gene from *V. cholerae* were used for constructing this tree. The trees were constructed by using maximum likelihood model with a bootstrap value of 1000 by using MEGAX. The scale bars in the trees indicated nucleotide substitutions per site for each tree separately. Partial sequence of 16S rRNA from *Bacillus* spp was used as outgroup in tree A and partial sequence of carbapenem resistance gene from *E. coli* was used as out group in tree (**b**), (**c**) and (**d**).

The phylogenetic analysis revealed that the partial amplicons of *qnr*B (468 bp) isolated from *E. coli* were closely related with the previously reported reference *qnr*B genes from *E. coli* (CP031833) and *Klebsiella pneumoniae* (EU127476) (Fig. 2 part a). The partial sequences of amplicons of *mcr*-1 gene (1561 bp) of *E. coli*, *V. cholerae* and *Salmonella* spp. clustered closely with reference *mcr*-1 genes of *E. coli* of accession number, MW836072, CP101213, and KY013597 (Fig. 2 part b). Further, the partial amplicons of *bla*_TEM_ (750 bp) encoded from the study isolates were closely related with each other and reference sequences of database. Study amplicons, TEM/JU/BD/22-1, TEM/JU/BD/22-5, TEM/JU/BD/22-6, TEM/JU/BD/22-8, and TEM/JU/BD/22-17 clustered closely with reference *bla*_TEM_ ON221404, ON221405, and AB700703 (Fig. 2 part c). Partial amplicons of resistance gene against tetracycline (*tet*) (211 bp) isolated from pathogenic isolates of *E. coli* of this study were closely related with the reference *tet* (36) (NG048131) and AJ514254 (Fig. 2 part d).

### Mutational analysis of *bla*_TEM_

After aligning the sequences against reference sequences of respective resistance genes we searched for the presence of mutations. However, we did not find any significant mutations in the *tet*, *qnrB* and *mcr-1* genes. We found significant mutations in the *bla*_TEM_ resistance genes. In most of the study strains the amino acids up-to 158 residues were highly similar with the reference sequences. Notable substitution point mutations were found in TEM/JU/BD/22–4 and TEM/JU/BD/22–9. In TEM/JU/BD/22–4, substitution mutations were found at positions 159 (Ser → Ile), 169 (Phe → Leu), 174 (Val → Arg), and 177 (Cys → Asp). In TEM/JU/BD/22–9, two substitution mutations were seen at positions 187 (Thr → Lys) and 188 (Glu → Asn).

## Discussion

According to the world health organization diarrhea is the 2^nd^ major cause of mortality and the leading cause of malnutrition among children under five years^1–3^. The health burden of diarrheal diseases is severe in developing countries like Bangladesh^3^. The circulation of antimicrobial resistance strains of these bacterial pathogens is intensifying the health burden of diarrheal disease in Bangladesh^13–15^.

We detected significantly higher prevalence of bacterial infection (63%) than any other previous reports from Bangladesh^1,3,9^. Further, the proportionate incidence of diverse pathogens was alarming. We found *E. coli* (29%) in higher frequency followed by *Shigella* spp. (17%) and *V. cholerae* (13%). The diversity of bacterial pathogens associated with diarrhea in children are similar with previous studies^3,9^. The higher prevalence of *Shigella* spp. requires further investigation and *V. cholerae* may indicate the constant presence of cholera endemic in these regions. Findings are in good agreement with previous studies in Bangladesh, China, India, Iran and WHO data from developing countries^2,8,15–25^.

We found significantly diverse pathogens in children with diarrhea. Mixed infection with rotavirus, norovirus, adenovirus and human bocavirus was simultaneously found in the positive specimens of bacterial infection. Dual infection by rotavirus and *E. coli* was associated with (15%) of the cases followed by *V. cholerae* and *Shigella* spp. (7.7%). Further, we detected that 31% specimens had co-infection with two pathogens and 12% with three pathogens. Few previous studies have also reported the presence of multiple pathogens in the same specimens in Bangladesh. Our findings are in good similarity with the previous findings in Savar and Tangail in Bangladesh^3, 9,18^.

Association of coinfection with the clinical symptoms were also analyzed in this study. We found that presence of coinfection was significantly associated with higher odds of diarrhea, vomiting, dehydration and abdominal pain in children under five. The presence of multiple pathogens in a single patient makes it difficult to intervene or treat the conditions earlier. Among the co-infected samples, we could not determine which pathogen infected earlier. However, the presence of coinfection was associated with severe and chronic outcomes in children under five. These findings are supported by the data of previous studies^3,5,8,9,18^. These findings also indicated that the existing hygienic conditions or setting of the study area favored the spread of multiple pathogens associated with diarrhea.

Significantly higher prevalence of antimicrobial resistance (AMR) pathogens from family *Enterobacteriaceae* was confirmed by the disk diffusion method in this study. About 60% of the isolates of *E. coli*, *V. cholerae*, *Salmonella* spp., and *Shigella* spp. were resistance against ceftriaxone, erythromycin, norfloxacin, ciprofloxacin and tetracycline. These antibiotics are most commonly used in treatment of diarrheal diseases caused by *Enterobacteriaceae* and supported by the CLSI guidelines. These findings are supported by previous studies in Savar and Tangail Bangladesh and other developing countries^3,15–21,24–27^. However, we found higher incidence of antimicrobial resistance pathogens than the previous studies in Tangail^18^ and Savar^3^, which suggests that the problem of AMR is increasing rapidly and widely in Bangladesh. These findings suggest that use of any of these antibiotics against *E coli*, *V cholerae*, *Salmonella* spp. or *Shigella* spp. will be less effective option in treatment, which is also supported by previous studies^24,25^. Except imipenem, meropenem and colistin, significant resistance activity was found in all of the detected bacterial pathogens among children. Among the tested antibiotics, the highest frequency (nearly 70%) of sensitivity was found against imipenem and meropenem. Though colistin is not used clinically in treatment of diarrhea caused by *Enterobacteriaceae*, we found higher prevalence of colistin resistance pathogens. This outcome is supported by previous studies in Bangladesh^3,18,27,28^.

The multidrug resistance (MDR) profiling of the isolated pathogens was alarming. Significantly higher prevalence of MDR was detected (about 15%) among the isolates of *E. coli*, *V. cholerae* and *Shigella* spp. against several antibiotics including ceftriaxone, erythromycin, norfloxacin and chloramphenicol. This finding is supported by previous studies in Bangladesh, China and Iran^3,14,17, 18,25–28^. However, the report of MDR from pathogenic bacteria isolated from children with diarrhea is less available in Bangladesh. In addition, higher prevalence of MDR against these important and commonly used antibiotics is staggering. The prevalence of MDR strains of pathogenic bacteria might be a significant cause of severe and prolonged health outcomes among children with diarrhea in Bangladesh. The rapid and uncontrolled increase in use of antibiotics as human treatment and animal feed and treatment options has significantly contributed to the rise and spread of MDR, which is supported by the previous studies^28^.

Integration of molecular characteristics and epidemiological data can contribute significantly in tracing the origin and transmission of AMR and MDR strains of pathogenic *E. coli*, *V. cholerae*, *Salmonella* spp. and *Shigella* spp. Which will ultimately aid in tracing the sources of infection and contribute in reduction of health burden among children. Antibiotic resistance genes against different group of antibiotics were found among the isolates of *E. coli*, *V. cholerae*, *Salmonella* spp. and *Shigella* spp. Resistance genes against ciprofloxacin (*qnrB*), cotrimoxazole (*sxt*) and colistin (*mcr*-1) were found in lower frequency among the isolates of *E. coli* (⁓10%), *V. cholerae* (⁓13%), *Salmonella* spp. (⁓14%) and *Shigella* spp. (⁓15%). Antibiotic resistance gene *bla*_TEM_ was found in highest frequency among the isolates of *Salmonella* spp. (45%) followed by *E. coli* (36%)*,* and *V. cholerae* (36%)*.* Further, *tet*A gene was detected among 18–27% isolates of these enteropathogenes. These findings are in good agreement with previous studies in Bangladesh^3, 18^. However, the diversity and prevalence of resistance genes were significantly higher in this study than previous reports. Further, we found that the presence of resistance genes in pathogenic *E. coli*, *V cholerae*, *Salmonella* spp., *Shigella* spp. were significantly associated with the phenotypic resistance properties against ceftriaxone, cotrimoxazole, ciprofloxacin, tetracycline and colistin. The findings of these study are supported by the previous studies in Bangladesh and China^3,14,17–20,26–28^. This is one of the first study to report the association between phenotypic and genotypic resistance of bacterial pathogens in Bangladesh. These findings are alarming for the pediatric health in Bangladesh as these bacterial pathogens contribute for a significant number of cases. Both AMR and MDR pathogens with the presence and association of resistance genes with treatment failure among the children with diarrhea require extensive studies in future.

The prevalence of resistance *E coli, V cholerae*, *Salmonella* spp. and *Shigella* spp. were higher in this study than previous studies in Bangladesh, India, Nigeria, Pakistan and Iran^13,16,18,23–25^. This phenotypic resistance and presence of resistance genes indicate probable continuous present of AMR pathogens among children in Bangladesh. It is established that misuse and over use of antibiotics have contributed to the origin and spread of resistance properties among pathogens. Importance should be given to appropriate diagnosis of pathogens before suggesting and use of antibiotics in children with diarrhea. Strict and widespread public health surveillance, education and policy are required to prevent further misuse and overuse of antibiotics in Bangladesh.

Findings from this study will add knowledge in the pathogenic diversity of children with diarrhea. Further, integrated approach to determine the prevalence of antimicrobial resistance and multidrug resistance isolates added significant information in the field. This study will contribute in determining and understanding the actual scenario and causes of antimicrobial resistance problem and their molecular elements, which will aid in accurate diagnosis and treatment of diarrheal patients in Bangladesh.

## Conclusion

We detected high prevalence of *E coli, V cholerae*, *Salmonella* spp. and *Shigella* spp. among children with diarrhea in Bangladesh. Co-infection of viruses was also found at higher frequency among the children. Further, we detected higher incidence of antimicrobial and multidrug resistance isolates among these pathogenic bacteria in children with diarrhea. Findings from this study also suggested that presence of co-infection increased the severity and complications of the disease among children. Findings from this study will contribute in policy making to understand and reduce the health burden associated with antimicrobial and multidrug resistance enteropathogens.

## Methods

### Ethical approval

The ethical clearance was obtained from the Biosafety, Biosecurity & Ethical Committee at the Jahangirnagar University. The informed consent was taken from the participants before taking the survey.

### Method guidelines

The authors confirm that all the methods were performed in accordance with the relevant guidelines and regulations. All methods were performed in accordance with the our previously published articles^3,5,7,8^. For bacterial culture and antibiotic sensitivity test, guidelines from ATCC (https://www.atcc.org/) and CLSI (https://clsi.org/) were followed, respectively.

### Study population and fecal specimens

A total of 404 fecal specimens were collected from patients with diarrheal diseases from 3 clinics of two different localities (Savar and Tangail) in Bangladesh through January 2019 to December 2021. Samples were collected from Enam Medical College (135 of 404) and Dip Clinics (125 of 404) in Savar and Kumuduni Women's Medical College (144 of 404) in Tangail. Convenience sampling method was used to enroll the participants. Epidemiological and demographic data were collected from children aged below 18 years with acute gastroenteritis. The participants of this study were divided into seven age groups including the range in months, 1–3, 4–11, 12–23, 24–35, 36–47, 48- 60 and > 60 (Table 1). Clinical symptoms data of children were analyzed. Informed consent was obtained from the parents of patients. Inclusion criteria to take samples were patients age below 18 years, have symptoms of diarrhea according to WHO definition, reported in the clinic’s outpatients, inpatients or emergency department. Exclusion criteria were patients aged above 18 years, did not report of their diarrheal cases in these clinics during the study period, missing demographic data, and having other health complications. One fecal specimen was collected from every participant. After collection, the specimens were stored at − 20 °C. Transportation of the fecal specimens were conducted by maintaining − 20 °C temperature in ice box. All the specimens were stored at − 20 °C after being transported under maintaining proper conditions^3^. All methods were performed in accordance with the our previously published articles^3,5,7,8^.

### Laboratory tests for *V.cholerae*, DEC, *Salmonella* spp., and *Shigella* spp

Colony characterization of bacterial pathogens were performed on specific selective agar media. MacConkey Agar, Thiosulphate-Citrate-Bile Salt Sucrose (TCBS) Agar, and Salmonella Shigella (SS) Agar media (HIMEDIA, India) were utilized aseptically to isolate and identify diarrheagenic *E. coli* (DEC)*, V.cholerae, Salmonella* spp., and *Shigella* spp. Eosin Methylene Blue (EMB) Agar (HIMEDIA, India) for *E.coli* and Xylose Lysine Deoxycholate (XLD) Agar (HIMEDIA, India) for *Salmonella* spp. and *Shigella* spp. were used for further identification^3,9^.

### Antibiotic susceptibility test of enteric bacterial pathogens

Antibiotic susceptibility test was performed by following the Kirby-Bauer Disk Diffusion Test and according to the CLSI guidelines^3,29^. Antibiotics of nine different groups were used, following the CLSI manual (Table 5). The susceptibility of the pathogens to each antibiotic was determined by measuring the zone diameter, which was further classified as resistance, intermediate, or susceptible according to the CLSI guidelines. *E. coli* ATCC 25922 strain was used concurrently as control to determine the validity of the test protocol.

**Table 5:** Performance standards for *E. coli*, *V. cholerae*, *Salmonella* spp. and *Shigella* spp. isolated from children with diarrhea to antimicrobial susceptibility testing performed in this study.

Antibiotics groups	Antibiotics	Interpretive categories and zone diameter breakpoints, nearest whole mm		
		S	I	R
Cephem	Ceftriaxone (CRO, 30μg)	≥ 26	23–25	≤ 22
Carbapenems	Imipenem (IPM, 10μg)	≥ 23	20–22	≤ 19
	Meropenem (MEM, 10μg)	≥ 23	20–22	≤ 19
Macrolides	Erythromycin (E, 10μg)	≥ 23	14–22	≤ 13
Tetracycline	Tetracycline (TE, 30μg)	≥ 15	12–14	≤ 11
	Doxycycline (DO, 30μg)	≥ 14	11–13	≤ 10
Quinolones	Ciprofloxacin (CIP, 10μg)	≥ 26	22–25	≤ 21
	Norfloxacin (NOR, 10μg)	≥ 17	13–16	≤ 12
Phenicol	Chloramphenicol (CL, 10μg)	≥ 18	13–17	≤ 12
Lipopeptide	Colistin (C, 10μg)			
Folate pathway antagonist	Cotrimoxazole (COT, 25μg)	≥ 16	11–15	≤ 10

*S* Sensitive, *I* Intermediate, *R* Resistance.

### Bacterial genome extraction and polymerase chain reaction

The bacterial genome was extracted by using the previously described boiled DNA method^3,30^. Further, genome of virus was isolated and purified (Supplementary material). The isolated bacteria were identified by using polymerase chain reaction (PCR). The following sequences of 16S rRNA primers (F- AGT TTG ATC CTG GCT CAG and R- ACC TTG TTA CGA CTT) were used for PCR reaction^3^. The PCR reaction mixture contained 12.5 µl of 2X master mix (GoTaq Green Master Mix, Promega, USA), 1 µl of forward (F) primer, 1 µl of reverse (R) primer, 6.5 µl of nuclease-free water, and 4 µl of template DNA (Eppendorf, Germany). The final volume of reaction mixture was 25 µl. The PCR reaction was conducted in a thermal cycler (2720 Thermal Cycler, Applied Biosystems, USA). The PCR reaction was performed at 94 °C for 5 min, followed by 35 cycles at 94 °C for 30 s, 53 °C for 30 s, and 72 °C for 60 s^3^. The details of the PCR conditions for the detection of antimicrobial resistance genes are provided in the supplementary material.

### PCR reaction to detect antimicrobial resistance genes

Primer pair, F- CATCTACCACTTCATAGGCAGC and R- CAGCTTAACTCACCAAGGAC were used for the amplification of *sxt* (242 base pairs) resistance gene. The PCR of *sxt* was conducted at 94 °C for 2 min 1 cycle, followed by 35 cycles of 94 °C for 1 min, 60.5 °C for 1 min, 72 °C for 1 min and a final extension at 72 °C for 10 min. For the amplification of *qnrA* (492 base pairs) primer pairs, F- GGATGCCAGTTTCGAGGA and R- TGCCAGGCACAGATCTTG were used in a thermal cycle at 94 °C for 5 min followed by 35 cycles at 94 °C for 1 min, 59 °C for 1 min, 72 °C for 1 min and final extension at 72 °C for 10 min. Primer pairs F-GATCGTGAAAGCCAGAAAGG and R-ACGATGCCTGGTAGTTGTCC were used for the amplification of *qnrB* (469 base pairs) resistance gene (Table 6). The PCR was conducted at 94 °C for 2 min followed by 35 cycles at 95 °C for 45 s, 53 °C for 45 s, 72 °C for 1 min and final extension at 72 °C for 5 min. For *bla*_*TEM*_ (750) resistance gene primers, F- TCGGGGAAATGTGCGCG and R-TGCTTAATCAGTGAGGACCC were used (Table 6). PCR was done at 94 °C for 7 min followed by 30 cycles at 94 °C for 30 s, 53 °C for 30 s, 72 °C for 30 s and final extension at 72 °C for 5 min. Primer pair, F- GCTCGGTCAGTCCGTTTGTTCTTG and R- GGATGAATGCGGTGCGGTCTT were used for *mcr-1*, colistin resistance genes at 93 °C for 3 min followed by 35 cycles at 93 °C for 15 s, 57 °C for 30 s, 68 °C for 70 s followed by final extension at 72 °C for 5 min. For molecular detection of *tet* genes, previously published articles were followed (Table 6). All the PCR products were kept at 4 °C.

**Table 6:** List of primers used in this study.

Target gene	Sequence 5′ → 3′	Product Size (bp)	T_a_ (°C)	References
16S rDNA	F- AGT TTG ATC CTG GCT CAG R- ACC TTG TTA CGA CTT	1484	53	Sharif et al.^3^
*sxt*	F- CATCTACCACTTCATAGGCAGC R- CAGCTTAACTCACCAAGGAC	242	60	Sharif et al.^3^
*mcr-1*	F- GCTCGGTCAGTCCGTTTGTTCTTG R- GGATGAATGCGGTGCGGTCTT	1561	57	Liu et al.^34^
*qnrA*	F- GGATGCCAGTTTCGAGGA R- TGCCAGGCACAGATCTTG	492	59	Sharif et al.^3^
*qnrB*	F-GATCGTGAAAGCCAGAAAGG R-ACGATGCCTGGTAGTTGTCC	469	53	Jacoby et al.^33^
*bla*_*TEM*_	F- TCGGGGAAATGTGCGCG R-TGCTTAATCAGTGAGGACCC	750	53	Gundran et al.^35^
*tetA*	F- CGCCTTTCCTTTGGGTTCTCTATATC R- CAGCCCACCGAGCACAGG	211	55	Sharif et al.^3^

*bp* base pairs, *T*_*a*_ Annealing temperature.

### Viral genome extraction

Total viral genome was extracted by using the Promega SVA total genome kit according to the manufacturer’s protocols (Promega, Madison, USA)^5,7,8^. Reverse transcriptase polymerase chain reaction method was performed to detect rotavirus and norovirus and polymerase chain reaction to detect adenovirus and human bocavirus. Molecular sequencing method was applied to confirm genotypic characteristics of viruses^5,7,8^.

### Agarose gel electrophoresis

The amplicons of the PCR were electrophoresed by using 1.5% agarose gel. Horizontal gel electrophoresis was run for 30 min^3^. Ladder DNA of 1 kilo base pair was used and specific amplicons were visualized by using the UV spectrophotometer (SPECORD-205, Analytik-Jena, Germany).

### Nucleotide sequence analysis

The nucleotide sequences of PCR amplicons (DNA) positive for *E. coli*, *V. cholerae*, *Salmonella* spp. and *Shigella* spp. were determined with the Big-Dye terminator cycle sequencing kit and an ABI Prism 310 Genetic Analyzer (Applied Biosystems Inc. Foster City, CA). The sequences were analyzed by using Chromas 2.6.5 (Technelysium, Helensvale, Australia). Sequence homology was confirmed by using the BLASTn (https://blast.ncbi.nlm.nih.gov/Blast.cgi) program. Multiple sequence alignment (MSA) was performed in the BioEdit 7.2.6 software using the ClustalW Multiple Alignment algorithm^3,31^.

### Phylogenetic analysis

Phylogenetic relationship and molecular evolutionary analysis of *qnrB, mcr-1, bla*_*TEM*_ and *tet* genes with the reference sequences were conducted by using MEGA 10.0 software^32^. Phylogenetic trees were built by using the Maximum Composite Likelihood (MCL) method^3,32^. Reference sequences were retrieved from the GenBank Database (https://www.ncbi.nlm.nih.gov/nucleotide/). The datasets generated and/or analyzed during the current study are available in the NCBI repository. The following reference strains were used for phylogenetic analysis of *qnrB* gene: MF999219, CP059125, CP051692, CP031833, EU127476, CP096261, OM975894, CP088121, CP032204, CP054227, CP032204 and MN200703 (outgroup). Reference strains for *bla*_*TEM*_ gene were: CP050932, CP048610, ON221405, ON221404, NG050233, AB700703, CP094365, CP095663, CP095655, CP095626, CP095618, KP853092, CP087666, CP095156, KT417856, KY739693 and KF268350 (outgroup). *mcr-1* gene were: KY013597, OM069356, OM069355, OM038692, OM038691, CP102678, OL504740, CP101861, CP030156, CP101391, CP101366, CP100031, CP100017, CP101213, CP099722, CP081339, CP065023, CP091559, CP091557, MW421457, MW719568, CP090269, MW836072 and KF268350 (outgroup). *tet* genes: NG_048131, AJ514254, CP049298, CP043636, CP104212, CP089096, CP040909, KJ797592, CP103193, AY265739, CP043637, CP043637, and KF268350 (outgroup). The nucleotide sequences generated in this study were submitted in the GenBank, NCBI under the submission ID, 2664914, 2664908, 2664900, and 2664883 and accession number OQ297026-OQ297034.

### Statistical analysis

We used percentage to present categorical variables and mean/ median for continuous variables. Inferential statistics (*p* value) was applied for the analysis. We calculated odds ratio (OR) of categorical variables by using two-tailed Chi-square or Fisher’s exact tests with 95% confidence intervals (CIs). For two-tailed tests *p* value < 0.05 were considered as significant. We performed data analysis by using Statistical Product and Service Solutions (SPSS v24.0) software (IBM, US).

### Supplementary Information

Supplementary Figures.

## Data Availability

All the data are available in this manuscript and supplementary materials. The nucleotide sequences generated in this study were submitted in the GenBank, NCBI under the submission ID, 2,664,914, 2,664,908, 2,664,900, and 2,664,883 and accession number OQ297026-OQ297034.
