# Supplementary material for: Multidrug resistance pattern and molecular epidemiology of pathogens among children with diarrhea in Bangladesh, 2019–2021
Source: Sci Rep. 2023 Aug 26;13:13975. doi: 10.1038/s41598-023-41174-6 (PMC10460387; doi:10.1038/s41598-023-41174-6)
Supplement: Supplementary file 1 — Supplementary Figures. [file 41598_2023_41174_MOESM1_ESM.docx]

**Figure i.** Proportionate incidence of diarrheagenic bacterial pathogens among children.


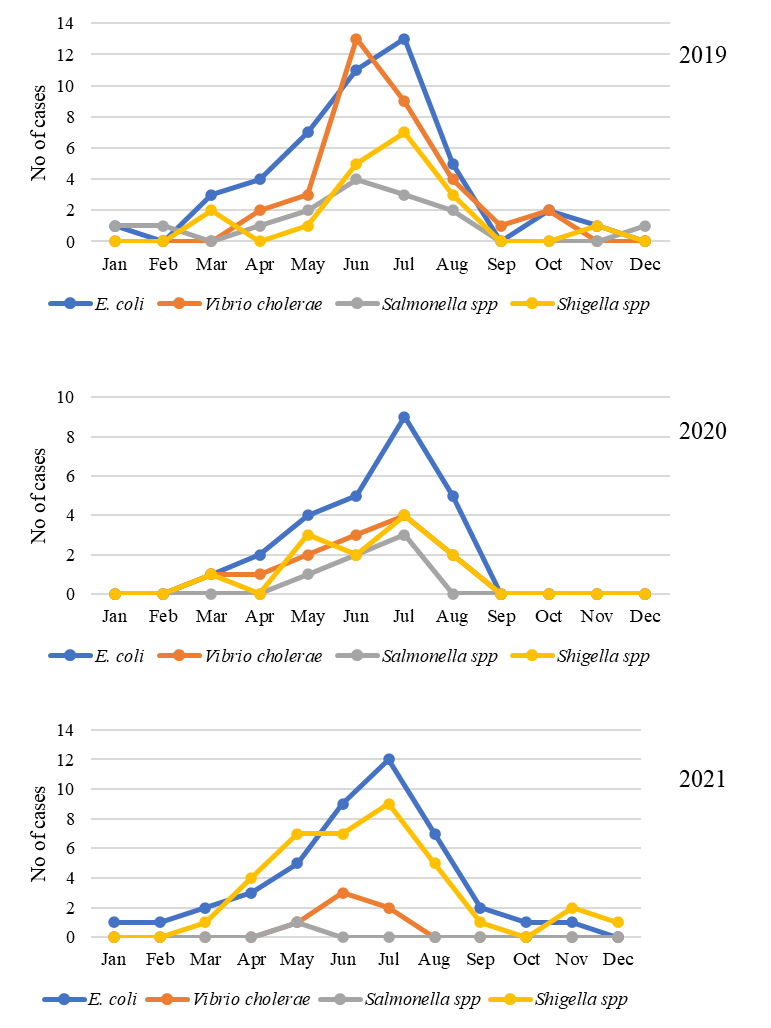


**Figure ii.** Seasonal distribution of bacterial pathogens isolated from children with diarrhea in Bangladesh.

**Figure iii.** Prevalence of mixed infection among children with diarrhea.

**Figure iv.** Frequency of clinical symptoms among patients infected with diarrheagenic pathogens.

**Figure v.** Duration of diarrhea among patients infected with bacterial pathogens.

**Figure vi.** Frequency of phenotypic and genotypic resistance properties of bacterial pathogens among patients with diarrhea.
